# Impact of a Whole-Food, High-Soluble Fiber Diet on the Gut–Muscle Axis in Aged Mice

**DOI:** 10.3390/nu16091323

**Published:** 2024-04-28

**Authors:** Roger A. Fielding, Michael S. Lustgarten

**Affiliations:** Nutrition, Exercise Physiology, and Sarcopenia Laboratory, Jean Mayer USDA Human Nutrition Research Center on Aging (HNRCA), Tufts University, Boston, MA 02111, USA; roger.fielding@tufts.edu

**Keywords:** soluble fiber, gut microbiome, short-chain fatty acids, skeletal muscle, weight loss, adiposity, aging, gut–muscle axis

## Abstract

Previous studies have identified a role for the gut microbiome and its metabolic products, short-chain fatty acids (SCFAs), in the maintenance of muscle mass and physical function (i.e., the gut–muscle axis), but interventions aimed at positively impacting the gut–muscle axis during aging are sparse. Gut bacteria ferment soluble fiber into SCFAs, and accordingly, to evaluate the impact of a high-soluble-fiber diet (HSFD) on the gut–muscle axis, we fed a whole-food, 3×-higher-soluble fiber-containing diet (relative to standard chow) to aged (98 weeks) C57BL/6J mice for 10 weeks. The HSFD significantly altered gut bacterial community structure and composition, but plasma SCFAs were not different, and a positive impact on muscle-related measures (when normalized to body weight) was not identified. However, when evaluating sex differences between dietary groups, female (but not male) HSFD-fed mice had significant increases for SCFAs, the quadriceps/body weight (BW) ratio, and treadmill work performance (distance run × BW), which suggests that an HSFD can positively impact the gut–muscle axis. In contrast, consistent effects in both male and female HSFD-fed mice included weight and fat loss, which suggests a positive role for an HSFD on the gut–adipose axis in aged mice.

## 1. Introduction

Skeletal muscle mass and physical function decline during aging, which puts older adults at a higher risk for frailty [1], falls and fracture [2,3], disability and hospitalization [4,5], and all-cause mortality [6]. Accordingly, the elucidation of mechanisms and interventions that positively impact muscle mass and physical function will be important for addressing the public health priority of improving healthspan-related outcomes in older adults.

The gut microbiome and its metabolic products are involved in mechanisms that impact skeletal muscle mass and physical function, which has been defined as the gut–muscle axis [7,8,9,10,11]. For example, muscle mass and physical function are reduced in germ-free [12,13] and in antibiotic-treated mice [14,15,16]. Investigating further, the SCFAs acetate, propionate, and butyrate are gut bacteria-derived metabolites that positively impact muscle mass and physical function in both young and aged animals [12,16,17,18,19,20]. When considering these findings, interventions that increase bacterial SCFA production may be a novel approach for resisting age-related changes to muscle-related measures.

Soluble fiber fermentation by gut bacteria results in the formation of acetate, propionate, and butyrate [21], and fecal SCFAs proportionally increase in response to high-soluble-fiber diets [22,23], which suggests that a high-soluble-fiber diet may positively impact the gut–muscle axis. In support of this, fecal and circulating levels of SCFAs, muscle mass, and aerobic exercise capacity were increased in young mice that were fed a higher-soluble-fiber diet [16]. In that study, standard chow was compared against a purified diet that contained cellulose as its fiber source, which is not fermented by gut bacteria to produce SCFAs [24]. In contrast, the impact of a whole-food HSFD (higher than standard chow) on muscle-related measures in aged mice has yet to be published. To address this knowledge gap, we fed a custom, whole-food HSFD to aged mice and evaluated the impact on gut microbiome composition, circulating SCFAs, and muscle-related measures, in comparison with age-matched mice fed standard chow.

## 2. Materials and Methods

### 2.1. Animals, Overview

Aged C57BL/6J male and female mice (91 weeks at the initial study assessment) were housed at the Jackson Laboratory (Bar Harbor, ME, USA), which additionally performed measurements of body composition, grip strength, and treadmill endurance capacity; harvested skeletal muscle; and collected stool and blood. The protocol (research question, study design) was created by the study PI (Michael Lustgarten), which was approved and conducted by Jackson Laboratory staff. Stool was collected, and body composition and physical function assessments were performed 7 weeks before initiating the dietary intervention. A total of 20 mice (10 males, 10 females) were randomly chosen (with an equal number of males and females in each dietary group) to consume the custom HSFD or standard chow (LabDiet 5K52), but three mice died in-between the period from randomization to HSFD initiation, thereby resulting in 8 mice (4 females, 4 males) that consumed the HSFD and 9 mice (4 females, 5 males) that consumed standard chow. A total of 20 mice were initially included based on sample size calculations derived from the effect of a higher-soluble-fiber-containing diet on fecal SCFAs in Okamoto et al. [16].

The study PI and Jackson Laboratory staff were aware of the group allocation at study initiation; only Jackson Laboratory staff had access to within-study data, which was provided to the study PI after study completion for use in statistical analyses. Mice were cohoused based on sex and diet: females and males were separately co-housed, and mice that consumed the control diet were housed in separate cages relative to mice that consumed the HSFD. After 2 and 6 weeks on the study diets, body composition and grip strength assessments were performed; feces was collected at the week-6 assessment. Treadmill endurance capacity was assessed 7 to 8 weeks after initiation of the dietary intervention. Mice remained on their respective diets until sacrifice (10 weeks), where muscle mass was harvested and feces and blood were collected. Humane endpoints were not established for this study, as we aimed to keep the mice on the study diets for 10 weeks. The experimental timeline is shown in Supplementary Figure S1.

### 2.2. Diets

To prepare the HSFD, air- and freeze-dried foods (broccoli and blackberry, beets, carrots, and red bell peppers) were obtained from Van Drunen Farms (Momence, IL, USA). Walnuts were obtained from the California Walnut Board and Commission (Folsom, CA, USA). Foods were then shipped to LabDiet (St. Louis, MO, USA), which, in combination with fish meal and a vitamin–mineral mix (both are also found in standard chow), pelleted the HSFD for consumption. Per kg, individual food amounts included broccoli, 336 g; purple beet, 167.04 g; carrot, 149 g; red bell pepper, 125 g; blackberry, 93 g; walnut, 57 g; fish meal, 30 g; and micronutrient mix, 42.97 g. This diet was designated as 5G4J-full nutrient composition and is shown in Supplementary Table S1. 5K52 (“control diet”) and 5G4J (“HSFD”) were fed to aged mice ad libitum.

### 2.3. Diet Composition Analysis

To evaluate diet composition, samples (10 g) of the custom and control diets were sent to Eurofins Microbiology Laboratories (North Kingstown, RI, USA). The caloric content (kcal/kg) was lower (3013 vs. 3296), whereas the soluble fiber was 3-fold higher (6.93 vs. 2.29 g/100 g) for the HSFD, when compared with the control diet. The diets were designed to be similar in terms of protein, carbohydrate, and fat, but compositional analysis showed that the protein content was 5% lower (14.4 vs. 19.6%), whereas the carbohydrate content was 4% higher for the HSFD, when compared with the control diet (61.7 vs. 57.3%). The diet composition for both diets is shown in Supplementary Table S2.

### 2.4. Stool DNA Extraction, High-Throughput Sequencing, and Taxonomy Assignment

Stool was collected from 17 mice (9 controls, 8 HSFD) seven weeks prior to starting this study, and after 6 weeks on the study diets; 15 stool samples (8 controls, 7 HSFD) were available at the week-10 assessment. DNA extraction and high-throughput sequencing were performed on fecal samples based on the method of Thorpe et al. [25]. DNA was extracted from stool with the QIAamp PowerFecal Pro DNA kit (QIAGEN, Germantown MD, USA). An amount of 250 mg of frozen stool was suspended in 800 μL of CD1 buffer in PowerBead Pro tubes (QIAGEN) in accordance with the kit protocol. The sample was then incubated at 65 °C for 10 min after which the manufacturer’s protocol was resumed. Samples were subjected to 5 min of bead-beating on a Vortex Genie (Scientific Industries, Inc., Bohemia, NY, USA) with a MoBio adapter (QIAGEN), and DNA was eluted in 50 μL of buffer. If the DNA concentration on a Nanodrop 2000 (Marshall Scientific, Hampton, NH, USA) was <10 μg/mL, the extraction was repeated. A portion of the extracted DNA was removed for 16S rDNA amplicon generation.

Amplicons of the V4 region of bacterial 16S rDNA were generated from extracted DNA as described by Caporaso et al. [26]. Triplicate PCR amplifications on 50 ng of DNA were performed using a 5′ HotStarTaq Master Mix kit (Thermo Fisher, Waltham, MA, USA) with the following conditions: 95 °C for 5 min, 30 cycles at 95 °C for 30 s, 53 °C for 45 s, 72 °C for 45 s, and a final extension at 72 °C for 10 min. Amplicon DNA concentrations were determined by Quant-iT assay (Invitrogen, Carlsbad, CA, USA), pooled in equimolar concentrations, purified (Qiaquick PCR Purification Kit, QIAGEN), and eluted from Agencourt Ampure XP beads (Beckman-Coulter, Brea, CA, USA). Amplicon pools were sequenced on an Illumina MiSeq (Illumina, San Diego, CA, USA) at the Tufts University Core Facility with a standard 250 bp paired-end Illumina protocol.

Sequencing results for the 16S amplicon libraries were processed using QIIME 2 (https://qiime2.org/). DADA2 (https://github.com/benjjneb/dada2) was used for read-joining and generation of the representative sequences. Taxonomy was assigned to the representative sequences with the q2-feature-classifier that was trained on the V4 regions of the Greengenes 13_8 database with OTUs of 99% similarity.

### 2.5. Plasma SCFA and Clinical Chemistry Analyses

Two weeks after completion of study measures, euthanasia and retro-orbital blood collection were performed on 15 mice (8 controls, 7 HSFD) using NIH guidelines [27]. Blood was collected into an EDTA tube, followed by centrifugation at 14,000 rpm at 4 °C. Plasma was then removed and stored in a 1.5 mL Eppendorf tube at −80 °C until analysis. Plasma levels of SCFAs were quantified by the Mass Spectrometry Core at the Tufts HNRCA according to the procedure of Dei Cas et al. [28] with liquid chromatography–tandem mass spectrometry (7500 QTRAP LC/MS/MS System, AB SCIEX LLC, Framingham, MA, USA).

A 6-parameter plasma metabolic panel consisted of albumin, ALT, AST, BUN, glucose, and creatinine. Cholesterol and triglycerides were measured with a clinical chemistry analyzer (AU480 Clinical Chemistry Analyzer, Beckman Coulter, Inc., Brea, CA, USA) by the Nutrition Evaluation Laboratory (NEL) at the Tufts HNRCA according to the manufacturer’s documented SOPs for the 6-parameter panel, and with previously published enzymatic procedures [29,30,31,32], respectively. High-sensitivity C-reactive protein (hsCRP) was quantified by the NEL according to the method of Babson [33] with a solid-phase, two-site chemiluminescent immunometric assay (IMMULITE 2000, Siemens Healthcare Diagnostics, Los Angeles, CA, USA).

### 2.6. Body Composition Assessment and Measurement of Skeletal Muscle Mass

A period of 7 weeks before the dietary intervention, and after 2 and 6 weeks on the study diets, whole-body lean and fat mass was evaluated with quantitative magnetic resonance imaging by the Jackson Laboratory. Live mice were placed into a thin-walled plastic cylinder (4.7 cm ID, 0.15 cm thick), where they were free to turn around but were limited to ∼4 cm vertical movements by a plastic insert. The plastic cylinder containing live mice was then placed into the qMRI machine (EchoMRI, Echo Medical Systems, Houston, TX, USA) for quantification of lean and fat mass. Measurements were completed after 2 min, and mice were returned to their home cage. Skeletal muscles (gastrocnemius, soleus, tibialis anterior, quadriceps, hamstrings) from both the lower hindlimbs were harvested at sacrifice (week 10) and weighed. The sum of each muscle from both legs was used for statistical analyses.

### 2.7. Physical Function Measures

Grip strength and treadmill endurance capacity were evaluated 6–7 weeks before initiating the dietary intervention, and after 6 and 7–8 weeks on the study diets, respectively, by the Jackson Laboratory. Grip strength was calculated as the average of all paws (in grams) during 3 trials with a BIOSEB grip strength meter (BIOSEB, Pinellas Park, FL, USA), as previously reported [34]. Treadmill endurance capacity was performed based on a revised version of our earlier protocol in young mice [35], including a longer acclimatization period (3 days) and running at a slower speed without sequential speed increments. On day 1, for familiarization, mice were placed on the treadmill without it being turned on. On day 2, mice were placed on the treadmill for a 20 min training session with a 0% incline and no shock stimulus. A cotton swab was used to prod the mice to run, if necessary. On day 3, the same protocol as day 2 was followed, but with the inclusion of a 10% incline. On day 4, mice were run until exhaustion using a 10% incline and the speed set to 14 m/s. Exhaustion was defined as the inability to run for 10 cumulative seconds despite mechanical prodding with a cotton swab, accumulating a total of 20 shocks at 0.3 mA, or an instance of 5 consecutive seconds of shock. Distance travelled in meters was recorded. Treadmill work was calculated as body weight multiplied by distance travelled.

### 2.8. Statistical Analysis

#### 2.8.1. α-, β-Diversity

α-diversity measures included evenness (how relatively abundant each of the species are) and the Shannon index (as a metric of species richness, i.e., how many species are observed in each sample). Raw α-diversity data were obtained from QIIME2, and between-group differences for the 6- and 10-week change from baseline were evaluated with the Mann–Whitey U test (*p* < 0.05).

β-diversity was evaluated with the web-based tool, MicrobiomeAnalyst (https://www.microbiomeanalyst.ca/) [36]. Data filtering settings included a low-count filter of 0, an 80% prevalence in samples, and 0 as the percentage to remove using the low-variance filter. Analysis of Bray–Curtis dissimilarities was performed with principal coordinate analysis using OTU relative abundance. Between-group community compositional differences were investigated with analysis of similarities (ANOSIM).

#### 2.8.2. Within- and between-Group Differences for Food and Energy Intake, Taxonomy, SCFAs and Clinical Chemistry Analytes, Body Weight and Composition, and Muscle-Related Measures

To identify taxonomic features differentially represented between groups, comparisons for phyla and genus were performed with linear discriminant effect size (LefSe) analysis, which is present within MicrobiomeAnalyst. LefSe ranks features by effect size (visualized via dot plot), putting features that explain most of the biological difference at the top [37]. The threshold for discriminating features was set at a logarithmic linear discriminant analysis (LDA) score > 2 in conjunction with a false discovery rate (FDR) ≤ 0.05.

Within- and between-group differences (pre-intervention control diet vs. HSFD; pre-intervention control diet and HSFD vs. Week 6 or Week 10; Week 10 control diet vs. HSFD) for phyla and genus were evaluated with the Mann–Whitney U test. Corresponding FDRs (839 comparisons) were calculated using the Benjamini–Hochberg method [38].

A two-sample *t*-test (*p* ≤ 0.05) was used (Microsoft Excel Version 16.84) to evaluate between-group (and sex-specific) differences for the following: weekly and cumulative (6 or 10 weeks) food and energy intake; the 2, 6, or 7–8 change from baseline for body weight and composition (lean and fat mass), grip strength, treadmill endurance capacity, and work; and week-10 differences for muscle mass, SCFAs, and clinical chemistry analytes.

## 3. Results

### 3.1. Daily Food and Energy Intake

**Figure 1:**
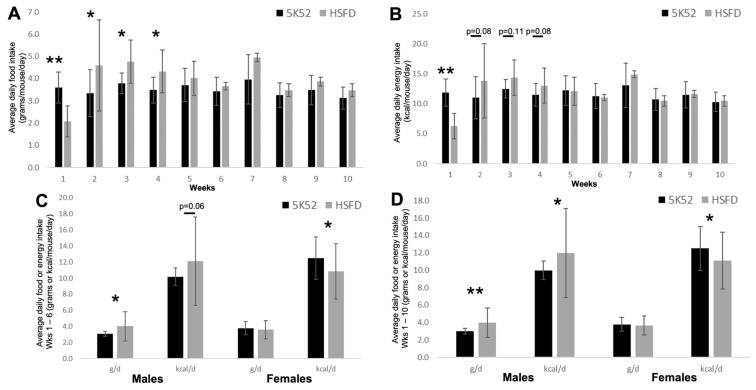
Average daily food intake (**A**), and average daily energy intake (**B**) in control-fed (black bars) and HSFD-fed (grey bars) mice. Average daily food or energy intake, Weeks (Wks) 1–6 (**C**) and 1–10 (**D**), separated by gender. Within (**C**) and (**D**), data for males and females are shown on the left and right of each respective image. * *p* ≤ 0.05; ** *p* ≤ 0.01.

When compared with controls, HSFD-fed mice ate significantly less food during the first week, and then ate significantly more food during weeks 2–4 (Figure 1A). Food intake was not different between groups during weeks 5–10. Dietary energy density (kcal/g food) was lower for the HSFD when compared with the control diet (3.013 vs. 3.296 kcal/g food), and accordingly, we then compared the daily energy intake (kcal/day) per mouse (Figure 1B).

The average daily kcal intake was significantly lower for HSFD-fed mice after 1 week on the diet, trending higher during weeks 2–4 (*p* = 0.08 to *p* = 0.11), but was not different between groups from weeks 5 to 10. Cumulative totals during weeks 1–6 for average daily food (control diet: 3.6 ± 0.8 vs. HSFD: 3.9 ± 1.5, *p* = 0.12) or energy intake (control diet: 11.7 ± 2.5 vs. 11.5 ± 4.5, *p* = 0.37) per mouse were not significantly different between groups. During weeks 1–10, HSFD-fed mice tended to eat more food/day than controls (3.8 ± 1.4 vs. 3.5 ± 0.8, *p* = 0.06), but the kcal/day/mouse was not different between groups (control diet: 11.7 ± 2.5 vs. HSFD: 11.5 ± 4.3, *p* = 0.42).

When comparing sex differences between groups (Figure 1C,D), male HSFD-fed mice ate significantly more food and close to significantly more kcal/day (*p* = 0.06) during weeks 1–6; during weeks 1–10, both food and daily energy intakes were significantly higher when compared with control-fed males. In contrast, the average daily food intake was not different during weeks 1–6 or 1–10 for HSFD-fed female mice, but the average daily energy intake was significantly lower at both time points when compared with sex-matched controls.

### 3.2. Altered Bacterial Community Structure in HSFD-Fed Mice

α-diversity measures (evenness, Shannon index) were not significantly different when comparing the 6- and 10-week change from baseline between dietary groups, or when comparing sex between groups at any time point (Supplementary Figure S2).

**Figure 2:**
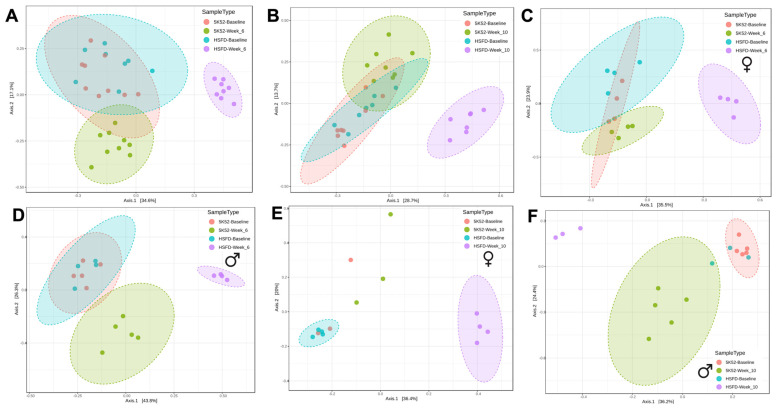
PCoA plots (Bray-Curtis) comparing bacterial β-diversity in HSFD- and control-fed mice for baseline with week 6 (**A**) and week 10 (**B**), in females (**C**,**E**) and males (**D**,**F**) at both time points, respectively. Orange, green, blue, and purple circles correspond to baseline controls, week-6 (or 10) controls, HSFD baseline, and HSFD week-6 (or 10).

In contrast, significant differences (ANOSIM *p* < 0.001) were identified when comparing between-group β-diversity at both the week-6 and week-10 evaluations (Figure 2A,B), and when analyzed separately based on sex (Figure 2C–F). Pre-dietary intervention, the bacterial community structure overlapped for mice randomized to the HSFD and controls, but after 6 and 10 weeks on their respective diets, a distinct separation occurred for HSFD-fed mice, including when separated based on sex.

#### 3.2.1. The Firmicutes/Bacteroidetes Ratio Is Reduced in HSFD-Fed Mice

**Figure 3:**
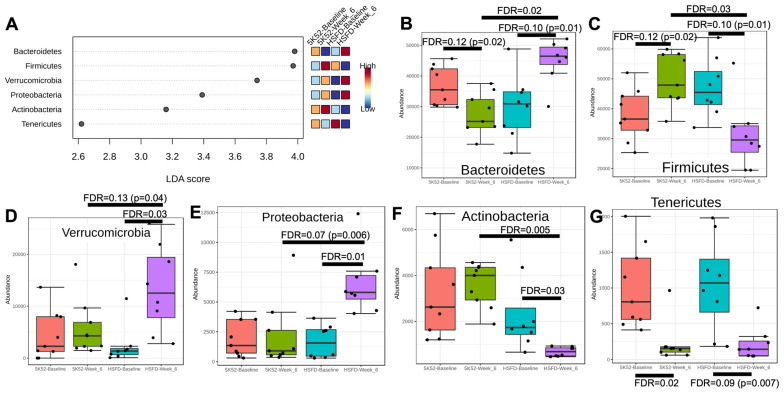
LEFSe dot plot (**A**) for phyla abundance when comparing week 6 with baseline in HSFD- and control-fed mice. Within- and between-group phyla abundance differences are shown in (**B**–**G**).

We then investigated taxonomic differences within and between groups: phyla abundance at the week-6 and week-10 assessments is visually represented in Supplementary Figure S3A,B. Pre-intervention, phyla abundance was not significantly different when comparing mice randomized to either dietary group (Supplementary Table S3). When comparing baseline and week-6 data, all six bacterial phyla had LDA scores > 2 (and FDR ≤ 0.05; Figure 3A). Within- and between-group phyla differences are shown in Figure 3B–G. Bacteroidetes were nominally reduced (*p* < 0.05, FDR > 0.05) within controls but were nominally and significantly increased within and between groups, respectively, at the 6-week time point for the HSFD. In contrast, data for Firmicutes were reversed: they were nominally increased and decreased within controls and the HSFD, and significantly lower between groups for the HSFD at the week-6 assessment. When evaluated as a combined metric, the pre-intervention Firmicutes/Bacteroidetes (F/B) ratio was not different when comparing mice randomized to the control diet and HSFD (1.1 ± 0.4 vs. 1.8 ± 1.0, *p* = 0.14), but after 6 weeks, the F/B ratio was nominally increased within controls (1.1 ± 0.4 vs. 2.3 ± 1.6, *p* = 0.01, FDR = 0.10), whereas it was significantly reduced within (1.8 ± 1.0 vs. 0.7 ± 0.2, *p* = 0.002, FDR = 0.03) and between groups for the HSFD (2.3 ± 1.6 vs. 0.7 ± 0.2, *p* = 8 × 10^−5^, FDR = 0.005).

Actinobacteria, Proteobacteria, and Verrucomicrobia were unchanged after 6 weeks within controls, but Actinobacteria were significantly decreased, whereas Proteobacteria and Verrucomicrobia increased within the HSFD. When comparing between-group data at the week-6 assessment, Actinobacteria were significantly reduced, whereas Proteobacteria and Verrucomicrobia were nominally increased for the HSFD. Tenericutes were significantly reduced within controls and nominally reduced within the HSFD; between-group differences at the 6-week time point were not identified.

**Figure 4:**
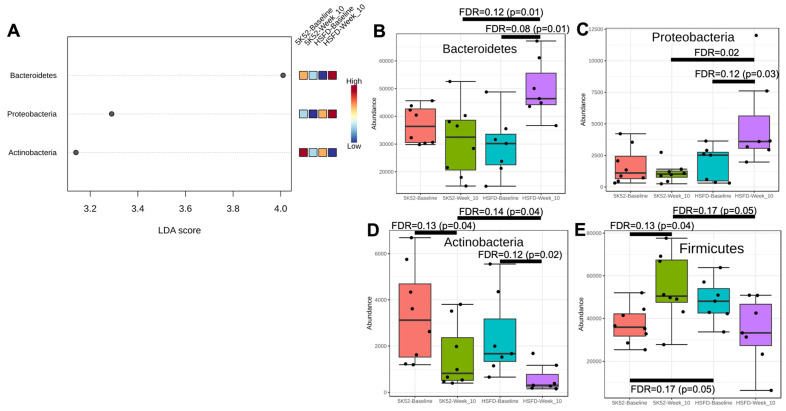
LEFSe dot plot (**A**) for phyla abundance when comparing comparing week 10 with baseline in HSFD- and control-fed mice. Within- and between-group phyla abundance differences are shown in (**B**–**E**).

After 10 weeks on the study diets, LDA scores > 2 (and FDR ≤ 0.05) were identified for three phyla: Bacteroidetes, Proteobacteria, and Actinobacteria (Figure 4A). Within- and between-group phyla abundance comparisons for baseline with week 10 are shown in Figure 4B–D. Bacteroidetes and Proteobacteria were unchanged after 10 weeks within controls but were nominally increased within the HSFD. Actinobacteria were nominally reduced within both the HSFD and controls. Although Firmicutes did not meet the LDA significance threshold, they were nominally higher within controls, and just outside of significance for a reduction within the HSFD (*p* = 0.07; Figure 4E).

Investigating further, the F/B ratio was not different when comparing week 10 against baseline for controls (2.3 ± 1.6 vs. 1.1 ± 0.4, *p* = 0.16), but it was nominally reduced within the HSFD (0.7 ± 0.4 vs. 2.0 ± 1.0, *p* = 0.01, FDR = 0.08). When comparing between-group differences at the week-10 assessment, Bacteroidetes and Proteobacteria were nominally and significantly higher, whereas Actinobacteria, Firmicutes, and the F/B ratio (2.3 ± 1.6 vs. 0.7 ± 0.4, *p* = 0.01, FDR = 0.12) were nominally lower for the HSFD.

Consistent phyla changes across both time points (weeks 6 and 10) include increases for Bacteroidetes and Proteobacteria within the HSFD, which were also higher for the HSFD when compared between groups. Conversely, Actinobacteria and F/B were reduced within and between groups for the HSFD.

#### 3.2.2. Reduced *Allobaculum Lactobacillus*, *Turicibacter*, and *Bifidobacteria* at Both Time Points within and between Groups for HSFD

**Figure 5:**
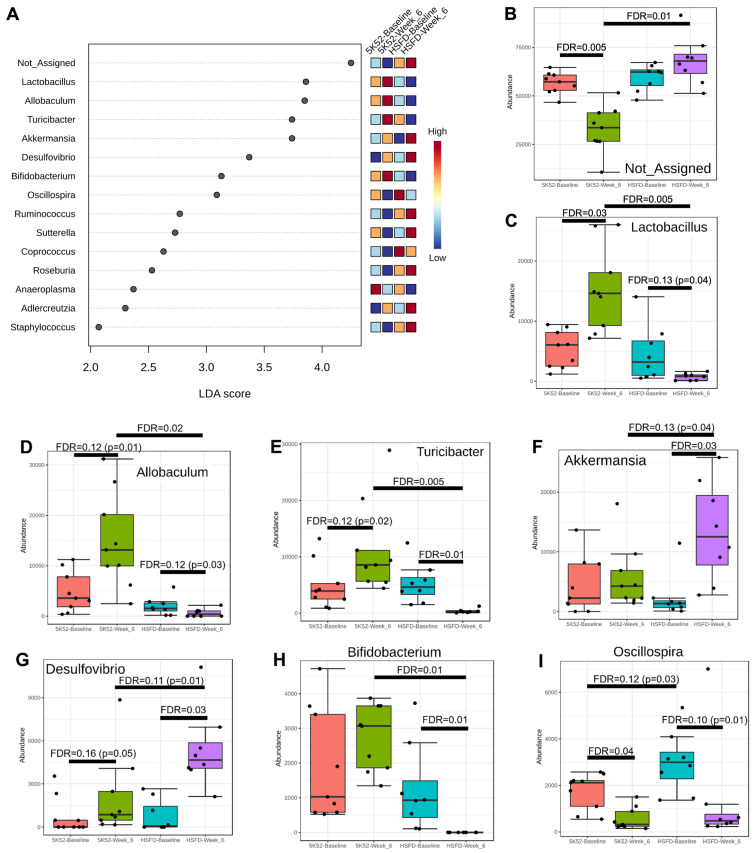

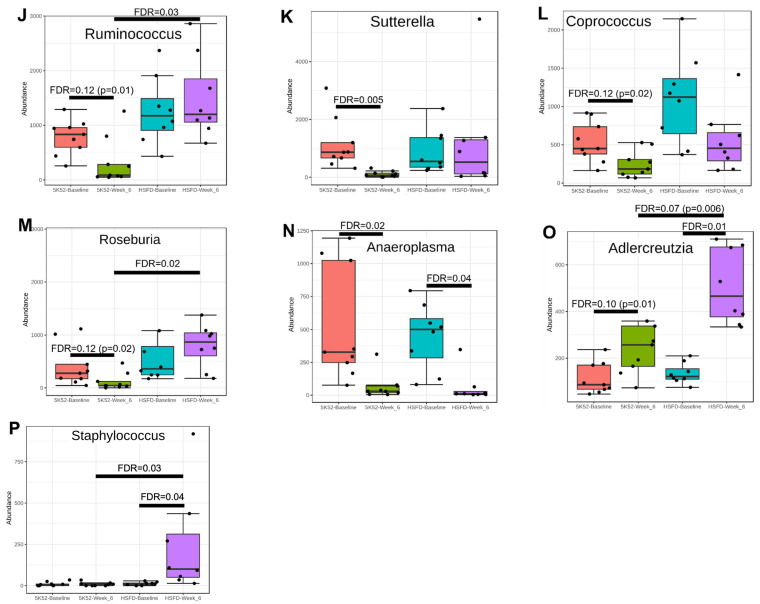
LEFSe dot plot (**A**) for genus abundance when comparing week 6 with baseline in HSFD- and control-fed mice. Within- and between-group genus abundance differences are shown in (**B**–**P**).

When including baseline and week-6 data, 15 bacterial genera had LDA scores > 2 (and FDR ≤ 0.05; Figure 5A). Within- and between-group genus abundance is compared in Figure 5B–P. Atop the list were unassigned bacteria, which comprised a majority of all genera in terms of abundance. After 6 weeks, unassigned bacteria significantly declined within controls, but not within the HSFD. Significant or nominal increases were identified within controls for *Lactobacillus*, *Allobaculum*, and *Turicibacter*, but in contrast, *Lactobacillus* and *Allobaculum* were nominally reduced, and *Turicibacter* was significantly reduced within the HSFD. *Akkermansia*, *Bifidobacterium*, and *Staphylococcus* were unchanged within controls, but significantly increased (*Akkermansia*, *Staphylococcus*) or decreased (*Bifidobacterium*) within the HSFD. Desulfovibrio and Adlercreutzia were nominally and significantly increased within controls and the HSFD, respectively. *Sutterella*, *Coprococcus*, *Ruminococcus*, and *Roseburia* were significantly or nominally reduced within controls, but were unchanged within the HSFD. Oscillospira and Anaeroplasma were significantly reduced within controls, and nominally and significantly reduced within the HSFD.

When comparing between-group differences at the week-6 assessment, unassigned bacteria, *Adlercreutzia*, *Desulfovibrio*, *Ruminococccus*, *Roseburia*, *Akkermansia,* and *Staphylococcus* were significantly or nominally higher, whereas *Lactobacillus*, *Allobaculum*, *Turicibacter*, and *Bifidobacterium* were significantly or nominally lower for the HSFD.

**Figure 6:**
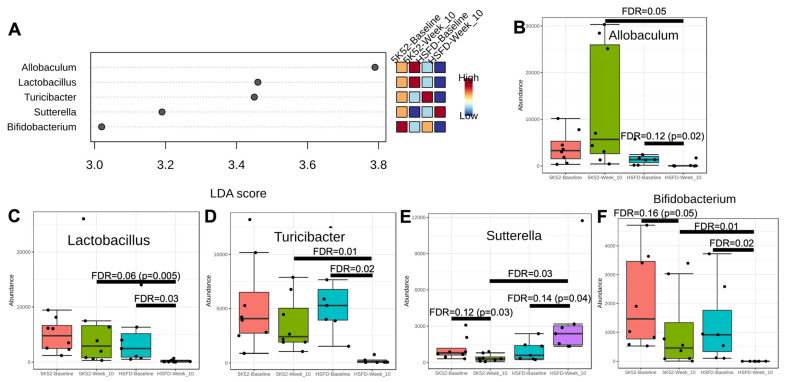
LEFSe dot plot (**A**) for genus abundance when comparing week 10 with baseline in HSFD- and control-fed mice. Within- and between-group genus abundance differences are shown in (**B**–**F**).

At the week-10 assessment, LDA scores > 2 (and FDR ≤ 0.05) were identified for five genera: *Allobaculum*, *Lactobacillus*, *Turicibacter*, *Sutterella*, and *Bifidobacterium* (Figure 6A–F). *Allobaculum*, *Lactobacillus*, and *Turicibacter* were unchanged within controls, but *Lactobacillus*, *Turicibacter*, and *Allobaculum* were significantly or nominally reduced within the HSFD. *Bifidobacterium* and *Sutterella* were nominally reduced within controls, but *Sutterella* was nominally increased, whereas *Bifidobacteria* was significantly decreased within the HSFD. When comparing between-group data at the week-10 assessment, *Allobaculum*, *Lactobacillus*, *Turicibacter*, and *Bifidobacterium* were significantly lower, whereas *Sutterella* was significantly higher in the HSFD.

Consistent genus changes across both time points (weeks 6 and 10) include reductions for *Allobaculum Lactobacillus*, *Turicibacter*, and *Bifidobacteria* within the HSFD, which were also lower for the HSFD when comparing between-group data. In contrast, *Sutterella* consistently declined within controls, whereas they were unchanged or increased within the HSFD.

#### 3.2.3. Sex Differences for Phyla and Genus within and between Groups

When comparing males and females separately within and between dietary groups, significant phyla and genus differences (FDR ≤ 0.05) were not identified, in part because of the small group sizes (week 6, males, females: 4 HSFD vs. 5 controls, 4 HSFD vs. 4 controls, respectively; week 10, males, females: 3 HSFD vs. 5 controls, 4 HSFD vs. 3 controls) and the high number of multiple comparisons (839). In contrast, nominally significant, sex-specific taxonomic differences were identified within and between groups at both time points (Supplementary Tables S4–S7). In terms of consistent differences based on sex or time, after 6 weeks on the study diets, Proteobacteria were increased within both male and female HSFD-fed mice, when compared with pre-intervention values. Between groups at the week-6 assessment, Actinobacteria and the F/B ratio were lower, whereas Bacteroidetes were higher within both male and female HSFD-fed mice, when compared with sex-matched controls. At both assessments (week 6 and week 10), Firmicutes increased within control-fed males and Tenericutes declined within HSFD-fed females when compared with pre-intervention values. No other consistent within- or between-group phyla differences at both time points were identified when comparing male and female HSFD-fed mice with sex-matched controls.

In terms of consistent genus-level changes across sex when comparing week-6 data with pre-intervention values (Supplementary Tables S8–S11), *Anaeroplasma* and *Sutterella* declined within both male and female control-fed mice. *Adlercreutzia*, *Corynebacterium*, and *Desulfovibrio* increased, whereas *Bifidobacterium* and *Turicibacter* decreased within both male and female HSFD-fed mice. Interestingly, *Sutterella* decreased within female but increased within male HSFD-fed mice. When comparing sex differences between dietary groups at the week-6 assessment, *Coprobacillus, Corynebacterium*, *unassigned bacteria*, and *Staphylococcus* were higher, whereas *Allobaculum*, *Bifidobacteria*, *Lactobacillus,* and *Turicibacter* were lower in both male and female HSFD-fed mice. Consistent genus-level differences were not identified at the week-10 assessment within both male or female mice for either dietary group when compared with pre-intervention values, but *Bifidobacterium* were lower for both male and female HSFD-fed mice when compared between groups with sex-matched controls.

### 3.3. Plasma SCFAs Are Increased in Female but Not Male HSFD-Fed Mice

**Figure 7:**
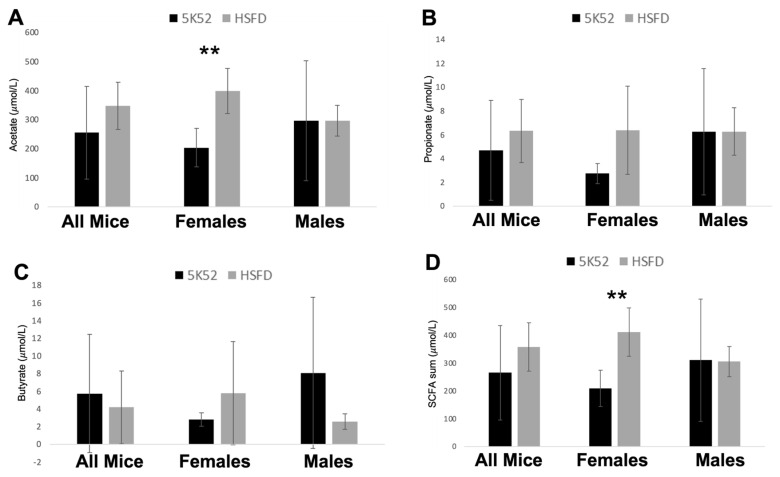
Plasma levels of the short-chain fatty acids (SCFAs), acetate (**A**), propionate (**B**), butyrate (**C**), and their sum (**D**) in all mice (left), females (middle) and males (right). Black bars, control diet; grey bars, HSFD. ** *p* ≤ 0.01.

Although plasma levels of acetate, propionate, butyrate, and their sum were not different when comparing HSFD- with control-fed mice, in contrast, acetate and the sum of these three SCFAs were significantly higher in female HSFD-fed mice, when compared with sex-matched controls (Figure 7A–D). SCFAs were not different when comparing between-group differences for male mice.

Plasma clinical chemistry measures, including glucose, BUN, AST, ALT, and albumin, were not different when analyzed between groups, or separately based on sex (Supplementary Table S12). hsCRP was below the detection limit (0.2 mg/L) in all mice. Plasma creatinine levels were significantly higher in HSFD-fed mice when compared with controls, an effect that was specific to male HSFD-fed mice. Triglycerides were not different between groups, but female HSFD-fed mice had significantly higher triglycerides when compared with sex-matched controls. These data suggest potentially worse kidney function (creatinine) and lipid metabolism (triglycerides) in HSFD-fed male and female mice, respectively. However, baseline creatinine and triglyceride levels were not measured, so it is unknown whether these biomarkers worsened or improved as a result of the HSFD over time.

### 3.4. Reduced Body Weight and Improved Body Composition in HSFD-Fed Mice

**Figure 8:**
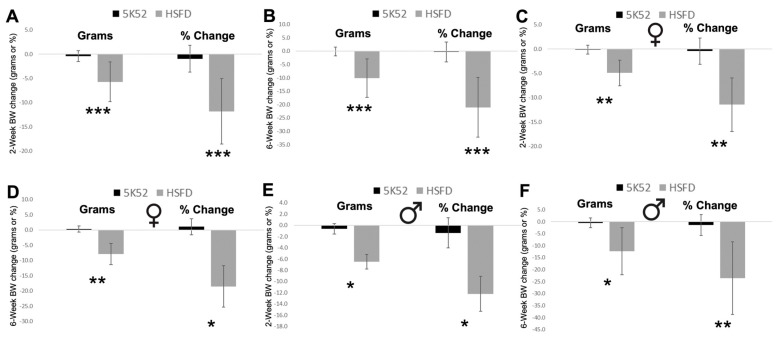
Body weight (BW) change from baseline, in grams or as percent change, after 2 weeks (**A**) and 6 weeks (**B**) in female (**C**,**D**) and male (**E**,**F**) mice are shown on the left and right of each image, respectively. Black bars, control diet; grey bars, HSFD. * *p* ≤ 0.05, ** *p* ≤ 0.01, *** *p* ≤ 0.001.

The 2- and 6-week changes from baseline for body weight, lean, and fat mass were each significantly reduced in HSFD-fed mice when compared with controls. Body weight was reduced by 5.7 and 10.1 g, which equates to 11.8 and 21.1% reductions, respectively (Figure 8A,B). When comparing sex differences between groups, the 2- and 6-week body weight change from baseline was significantly reduced by 4.9 and 7.9 g in HSFD-fed female mice (11,4%, 18.5% reductions) and by 6.5 and 12.3 g in HSFD-fed male mice (12.2%, 23.6% reductions; Figure 8C–F). In sum, despite the higher and lower average daily energy intakes for male and female HSFD-fed mice, respectively, body weight was consistently reduced, when compared with sex-matched controls.

**Figure 9:**
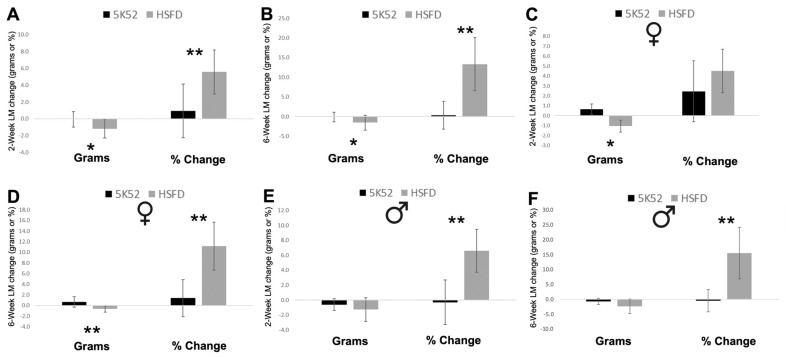
Lean mass (LM) change frorn baseline, in grams or as percent change, after 2 weeks (**A**) and 6 weeks (**B**) in females (**C**,**D**) and males (**E**,**F**) are shown on the left and right of each image, respectively. Black bars, control diet; grey bars, HSFD. * *p* ≤ 0.05, ** *p* ≤ 0.01.

**Figure 10:**
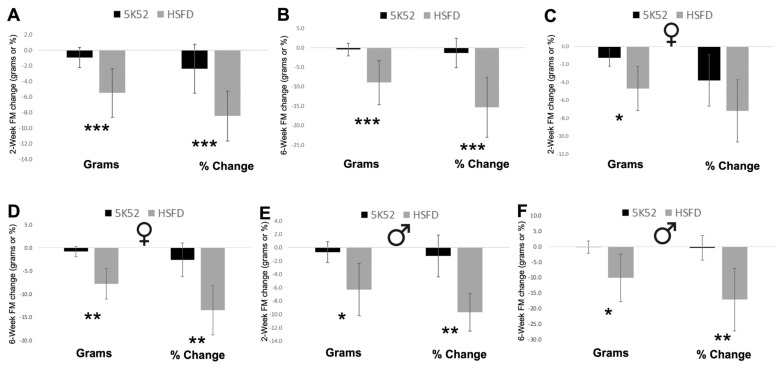
Fat mass (FM) change from baseline, in grams or as percent change, after 2 weeks (**A**) and 6 weeks (**B**) in females (**C**,**D**) and males (**E**,**F**) are shown on the left and right Of each image, respectively. Black bars, control diet; grey bars, HSFD. * *p* ≤ 0.05, ** *p* ≤ 0.01, *** *p* ≤ 0.001.

When subdividing body weight into body composition measures, the 2- and 6-week change for whole-body lean mass was reduced by 1.2 and 1.5 g, and whole-body fat mass was reduced by 5.5 and 9.0 g in HSFD-fed mice, when compared with control-fed mice (Figure 9A,B and Figure 10A,B). Despite these reductions, percent lean mass (lean mass divided by body weight) increased by 5.6% and 13.3%, whereas body fat percentage (fat mass divided by body weight) was reduced by 8.4 and 15.3% (Figure 9A,B and Figure 10A,B).

When comparing sex differences between groups (Figure 9C–F), the 2- and 6-week change for whole-body lean mass was significantly reduced by 1.1 and 0.6 g in female HSFD-fed mice, but it was not reduced for HSFD-fed male mice at either time point. The 2-week change for percent lean mass was not significantly different, but it was 11.2% increased after 6 weeks for HSFD-fed female mice. The 2- and 6-week change for percent lean mass significantly increased by 6.6 and 15.5% for HSFD-fed male mice when compared with sex-matched controls.

The 2- and 6-week change for fat mass was reduced by 4.7 and 7.8 g for female and by 6.3 and 10.2 g for male HSFD-fed mice, when compared with sex-matched control-fed mice (Figure 10C–F). The 2-week body fat percentage change was not different (*p* = 0.09) for HSFD-fed female mice, but it was significantly decreased by 13.5% after 6 weeks. The 2- and 6-week change for body fat percentage was significantly decreased by 9.7 and 17.2%, respectively, for HSFD-fed male mice. In sum, consumption of the HSFD by aged male and female mice reduced body weight and improved body composition, as evidenced by increased and decreased percentages of whole-body lean and fat mass, respectively.

### 3.5. Grip Strength Is Reduced in HSFD-Fed Mice, but Not When Divided by Body Weight

**Figure 11:**
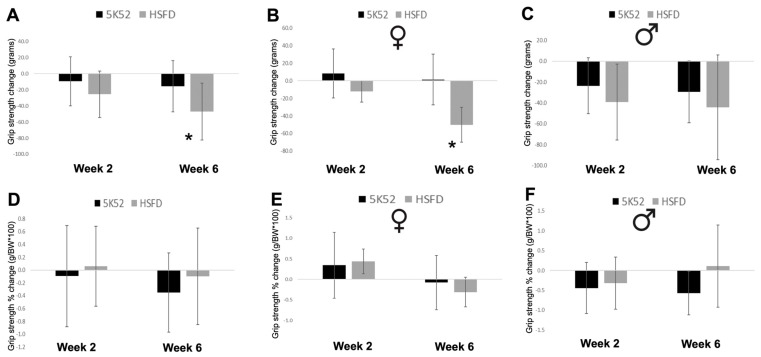
Grip strength change from baseline in grams (**A**), and when divided by body weight (grip strength/BW × 100%; **D**) after 2 and 6 weeks (left and right of each respective image) in females (**B**,**E**) and males (**C**,**F**). Black bars, control diet; grey bars, HSFD. * *p* ≤ 0.05.

The 2-week change from baseline for grip strength was not different between dietary groups, but the 6-week change was significantly reduced for HSFD-fed mice (Figure 11A). This effect was significant for female (but not male) HSFD-fed mice, which experienced a reduction for the 6-week grip strength change, when compared with controls (Figure 11B,C). However, when divided by body weight, the 2- and 6-week grip strength change was not significantly different when evaluated between groups (Figure 11D), or when analyzed separately based on sex (Figure 11E,F).

### 3.6. Improved Treadmill Endurance Capacity and Work, but Only in HSFD-Fed Female Mice

**Figure 12:**
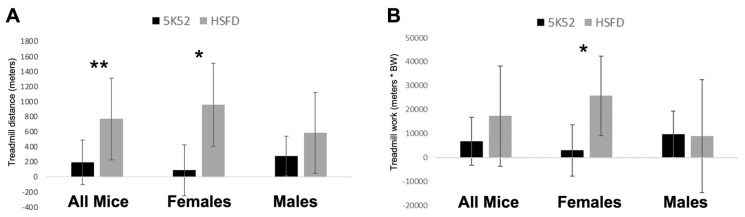
Treadmill endurance capacity (distance run, **A**), and treadmill work (distance run × BW, **B**) change from baseline, including data for all mice (left), females and males (middle, right, respectively) are shown. Black bars, control diet; grey bars, HSFD. * *p* ≤ 0.05, ** *p* ≤ 0.01.

The 7–8-week change from baseline for treadmill running distance was significantly longer for HSFD-fed mice when compared with control-fed mice (Figure 12A). Investigating further, this effect was significant for HSFD-fed female, but not male mice. When considering that body weight was reduced as a result of the HSFD, we then compared treadmill work performance (meters traveled ∗ BW; Figure 12B). Treadmill work performance was not significantly different between dietary groups, but female HSFD-fed mice performed more treadmill work than their sex-matched, control-fed counterparts. Between-group differences for male mice were not identified for treadmill work performance.

### 3.7. Muscle Mass Is Decreased in HSFD-Fed Mice, but Is Increased When Accounting for Body Weight

**Figure 13:**
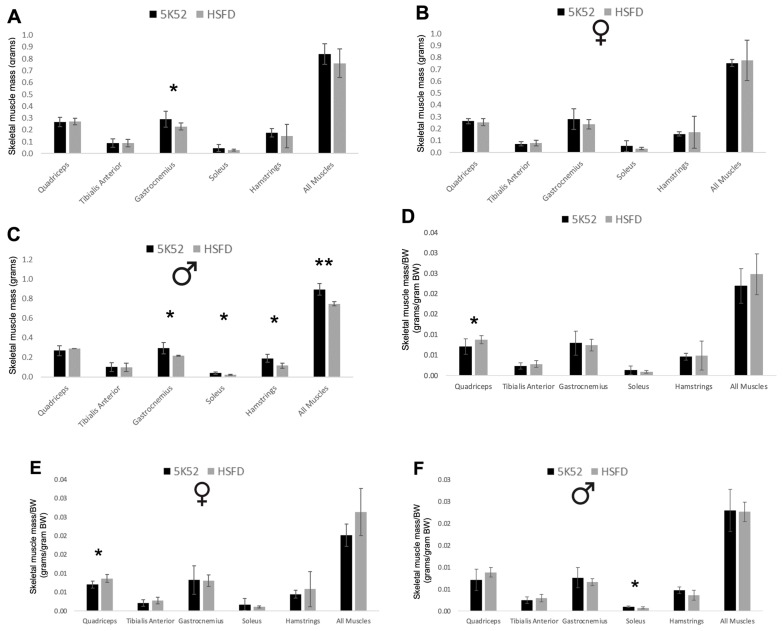
Skeletal muscle mass (**A**) and skeletal muscle mass divided by body weight (BW; **D**) in females (**B**,**E**), and in males (**C**,**F**). Black bars, control diet; grey bars, HSFD. * *p* ≤ 0.05, ** *p* ≤ 0.01.

Two weeks after treadmill testing, individual skeletal muscles, including the quadriceps, tibialis anterior, gastrocnemius, soleus, and hamstrings, were harvested from 15 mice (7 HSFD: 3 males, 4 females; 8 control diet: 5 males, 3 females). The gastrocnemius mass was significantly reduced in HSFD-mice, but the mass of the other muscles and their sum were not significantly different when comparing the two dietary groups (Figure 13A). When evaluating sex differences between groups, individual muscle masses and the sum of these five muscles were not different when comparing HSFD- with control-fed female mice (Figure 13B). In contrast, the masses of the gastrocnemius, soleus, and hamstrings and the five-muscle sum were significantly reduced in HSFD-fed male mice when compared with sex-matched controls (Figure 13C).

HSFD-fed mice experienced significant reductions in body weight, which could explain a corresponding decrease in muscle mass. To test this hypothesis, we evaluated between-group differences for muscle mass divided by BW. In contrast with the data for absolute levels of muscle mass, the quadriceps/BW ratio was significantly higher in HSFD-fed mice when compared with controls, an effect that was significant for female but not male mice (Figure 13D–F). Although the soleus/BW ratio was significantly reduced in HSFD-fed male mice, the five-muscle sum/BW ratio was not different when comparing between-group differences for male or female mice. In contrast with findings for absolute levels of muscle mass, these data suggest neutral (all mice, sum of the 5 muscles/BW), positive (increased quadriceps/BW in females), and negative (decreased soleus/BW in males) roles for the HSFD on the maintenance of muscle mass in aged mice.

## 4. Discussion

The primary goal of the present study was to evaluate the impact of a whole-food, higher-soluble-fiber diet (3× when compared with standard chow) on the gut–muscle axis in aged mice. The HSFD significantly altered gut bacterial community structure (β-diversity), and more specifically, within- and between-group differences for phyla and genus abundance were identified. Despite these alterations, plasma SCFAs were not higher in HSFD-fed mice, when compared with controls. In terms of the impact on muscle-related measures, when normalized to body weight, quadriceps mass was significantly increased, but the sum of five individual muscles, the grip strength, and the treadmill endurance capacity were not different between groups. Collectively, these data argue against the impact of a whole-food, high-soluble-fiber diet on the gut–muscle axis in aged mice.

However, when data were analyzed based on sex differences between groups, a positive impact for the HSFD on the gut–muscle axis was identified for female (but not male) mice. Although β-diversity was significantly different for both female and male HSFD-fed mice when compared with sex-matched controls, plasma SCFAs, the quadriceps/BW ratio, and treadmill work performance were significantly increased in female HSFD-fed mice when compared with control-fed females. In contrast, although the soleus/BW ratio was reduced, fecal SCFAs, the five-muscle mass sum/BW, grip strength/BW, and treadmill work performance were not different for HSFD-fed male mice, when compared with sex-matched controls.

The differential intake for male and female mice may have confounded a potential impact for the HSFD on outcome variables. HSFD-fed female mice ate a similar amount of food when compared with control-fed females, but because the HSFD had a lower caloric density, less kcal/d was consumed. In contrast, male HSFD-fed mice ate more food than male controls, thereby resulting in a higher daily energy intake. To address this disparity, future studies that standardize energy intake across groups may allow for a more accurate assessment for an HSFD’s potential impact on the gut–muscle axis in aged mice.

Regardless of energy intake, large reductions for body weight and fat mass were identified for both male and female HSFD-fed mice and when compared together between dietary groups, which suggests a role for an HSFD on the gut–adipose axis. In support of these findings, higher intakes of soluble fiber reduced body weight and fat mass in young mice [39,40,41]. In aged mice, dietary supplementation with the soluble fiber, inulin, reduced body weight and fat mass in 11- but not 26-month-old mice [40], which suggests that a whole-food-based, high-soluble-fiber diet may be a better approach for improving body weight and composition in aged mice. Moreover, findings in male HSFD-fed mice demonstrate that a caloric deficit is not necessary to improve body mass and composition during aging. Male HSFD-fed mice lost weight and reduced fat mass despite consuming more food and kcal/day than control-fed mice, which suggests a potential role for an increased thermic effect of food. A higher fiber intake has been shown to increase the dietary thermic effect [42], which is a potentially important finding because the thermic effect of food declines during aging [43]. Alternatively, it is possible that spontaneous locomotor activity (which was not measured in the present study) was increased in male HSFD-fed mice. Future studies aiming to investigate mechanisms that may underlie weight and fat loss in response to an increased energy intake on an HSFD are of interest.

Microbiome changes in HSFD-fed mice that may be involved in mechanisms related to weight and/or fat loss include within- and between-group reductions for F/B, *Allobaculum*, *Lactobacillus*, *Turicibacter*, and *Bifidobacteria*. In agreement with the F/B reduction in HSFD-fed mice, the F/B ratio was reduced in young mice fed a higher-soluble-fiber diet (chow vs. a cellulose-containing, purified diet) in conjunction with reduced fat mass [16]. In contrast, plasma SCFAs, muscle mass, and treadmill endurance capacity were increased in chow-fed mice in Okamoto et al. [16], whereas the impact of an HSFD on the gut–muscle axis in the present study was less clear. Moreover, the F/B ratio is elevated in obese mice when compared with lean mice [44] and is positively associated with a higher BMI in people [45,46], but it was reduced following supplementation with inulin in conjunction with reductions for body weight and fat mass [40]. Collectively, when considering that the F/B ratio increases during aging in mice [47] and in people [48], these data suggest that an HSFD may be an important approach for attenuating age-related changes for the F/B ratio, body weight, and fat mass.

*Allobaculum* (phyla: Firmicutes) is increased in mice on a high-fat diet in conjunction with increased body weight and fat mass [49]. High-fat diets use cellulose as the primary fiber source, which highlights a potential role for fermentable fiber on reductions for *Allobaculum*, adiposity, and body weight during aging. *Allobaculum* was reduced, whereas *Sutterella* (phyla: Proteobacteria) was increased in Okamoto et al. [16] and, in conjunction with similar findings in the present study, support a role for these bacteria on soluble-fiber-mediated mechanisms that may impact body weight and composition in mice. *Turicibacter* (phyla = Firmicutes) and fat mass increased in mice fed a high-fat, high-sugar “western diet” (WD, which also contained cellulose as the fiber source), but both were reduced when supplementing the WD with the soluble fiber, arabinoxylan [50]. Interestingly, in Kok et al. [51], *Turicibacter* was positively correlated with fat mass but were reduced in calorie-restricted (CR) mice, which suggests that the HSFD could act as a CR mimetic.

Reductions for *Lactobacillus* (phyla = Firmicutes) and *Bifidobacteria* (phyla = Actinobacteria) may have contributed to the HSFD’s inability to consistently impact the gut–muscle axis across both male and female mice. In support of this hypothesis, probiotic supplementation with *Lactobacillus* or *Bifidobacteria* species increased muscle mass, muscle strength, or endurance exercise capacity in mice and people [52,53,54,55]. Although *Lactobacillus* and *Bifidobacteria* species have been shown to reduce body weight and fat mass in mice [56,57], body weight and fat mass were reduced in HSFD-fed mice despite reductions for *Lactobacillus* and *Bifidobacteria*, which suggests that other bacteria contributed to these alterations. *Lactobacillus* was higher in mice fed a grain-based diet when compared to a purified, soluble-fiber-devoid diet [58]. Similarly, *Bifidobacteria* were increased in response to dietary supplementation with the wheat-bran-derived fiber, arabinoxylan, in conjunction with reduced fat mass [41]. Accordingly, when considering the previously published positive effects for *Lactobacillus* and *Bifidobacteria* on muscle- and fat-related measures, future iterations of the HSFD may include a mix of not only soluble-fiber-rich fruits and vegetables, but whole grains, too.

## 5. Limitations

Potential confounders for the impact of a higher-soluble-fiber diet on the gut–muscle axis include contributions from other plant components, including nitrate and ellagitannins. First, several studies have demonstrated that nitrate intake can improve muscle function and may impact muscle mass [59,60,61,62,63,64]. Nitrate-containing foods, including beets, broccoli, and carrots, were present in the HSFD, potentially resulting in a higher level of ingested nitrate for HSFD-fed mice, relative to the control diet. Second, the HSFD included walnuts—their ellagitannins [65] can be converted by gut microbiota into urolithin A [66], a metabolite that improves muscle strength, endurance, and exercise performance [67,68,69]. When considering that positive alterations to the gut–muscle axis were identified for HSFD-fed female mice, these effects may not be specific to soluble fiber, but to the combination of soluble fiber and the higher levels of nitrates and ellagitannins. Moreover, although the HSFD was initially designed to have a macronutrient composition that was equivalent to the control diet, after formulation, it contained 5% less protein by weight, which may have limited potential improvements to the gut–muscle axis in aged mice.

## 6. Conclusions

When including combined data for aged male and female mice, although an HSFD did not positively impact the gut–muscle axis, the differential intake based on sex may have affected these findings. Future studies with larger sample sizes to test potential sex-specific effects and that are matched for energy intake across groups would provide more insight. In contrast, consistent effects for the HSFD on reductions in body weight and fat mass in conjunction with alterations to gut microbiome community structure and composition provide evidence for an HSFD on the gut–adipose axis. When considering that global obesity rates have tripled within the past 40 years [70], findings from the present study may be an important step towards slowing this process [71].

## Data Availability

The data that support the findings of this study are available from the corresponding author upon reasonable request.

## Supplementary Materials

The following supporting information can be downloaded at https://www.mdpi.com/article/10.3390/nu16091323/s1. Figure S1: Experimental timeline; Figure S2: Week 6 and week 10 change from baseline for α-diversity measures in control- and HSFD-fed mice; Figure S3: Phyla relative abundance in HSFD- and control-fed mice for baseline vs. week 6 and week 10; Table S1: 5G4J (“HSFD”) formulation; Table S2: Diet composition, HSFD, and control diets; Table S3: Baseline phyla abundance comparisons, HSFD vs. control diet; Table S4: Within- and between-group phyla comparisons, week 6 vs. baseline, for male mice; Table S5: Within- and between-group phyla comparisons, week 6 vs. baseline, for female mice; Table S6: Within- and between-group phyla comparisons, week 10 vs. baseline, for male mice; Table S7: Within- and between-group phyla comparisons, week 10 vs. baseline, for female mice; Table S8: Within- and between-group genus comparisons, week 6 vs. baseline, for male mice; Table S9: Within- and between-group genus comparisons, week 6 vs. baseline, for female mice; Table S10: Within- and between-group genus comparisons, week 10 vs. baseline, for male mice; Table S11: Within- and between-group genus comparisons, week 10 vs. baseline, for female mice; Table S12: Clinical chemistry comparisons, HSFD vs. control diet.
