# Supplementary material for: Impact of a Whole-Food, High-Soluble Fiber Diet on the Gut–Muscle Axis in Aged Mice
Source: Nutrients. 2024 Apr 28;16(9):1323. doi: 10.3390/nu16091323 (PMC11085703; doi:10.3390/nu16091323)

Supplementary Figure S1 Experimental Timeline: 7-weeks before the dietary intervention, body composition, grip strength, and treadmill endurance capacity were assessed, and feces was collected. 2- and 6-weeks after initiating the dietary intervention, body composition and grip strength were evaluated; feces was collected at the week-6 time point. Treadmill endurance capacity was quantified 7 - 8 weeks after initiating the dietary intervention. After 10 weeks on the study diets, skeletal muscle mass was harvested, feces and blood were collected.

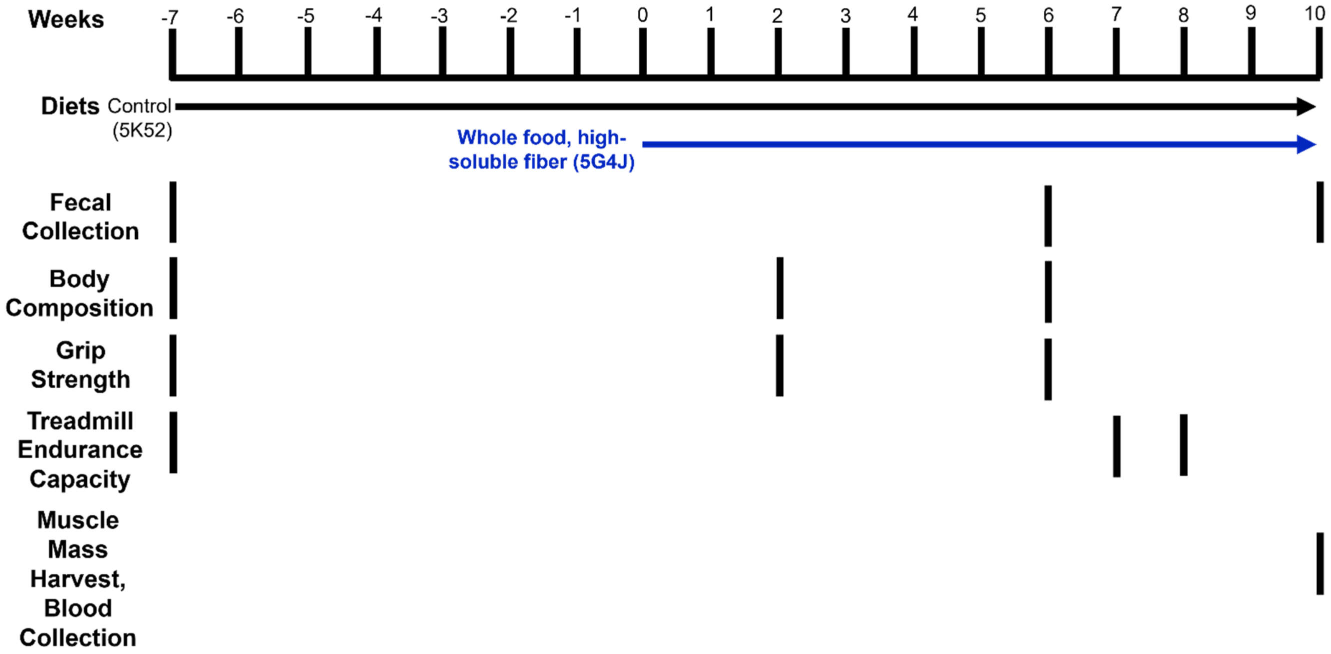

Supplementary Figure S2 Week 6 and Week 10 change from baseline for α-diversity measures, including evenness (A, B) and the Shannon index (C, D), respectively. Between-group data for all mice (blue, orange bars), females (grey, yellow), and males (light blue, green) is shown.

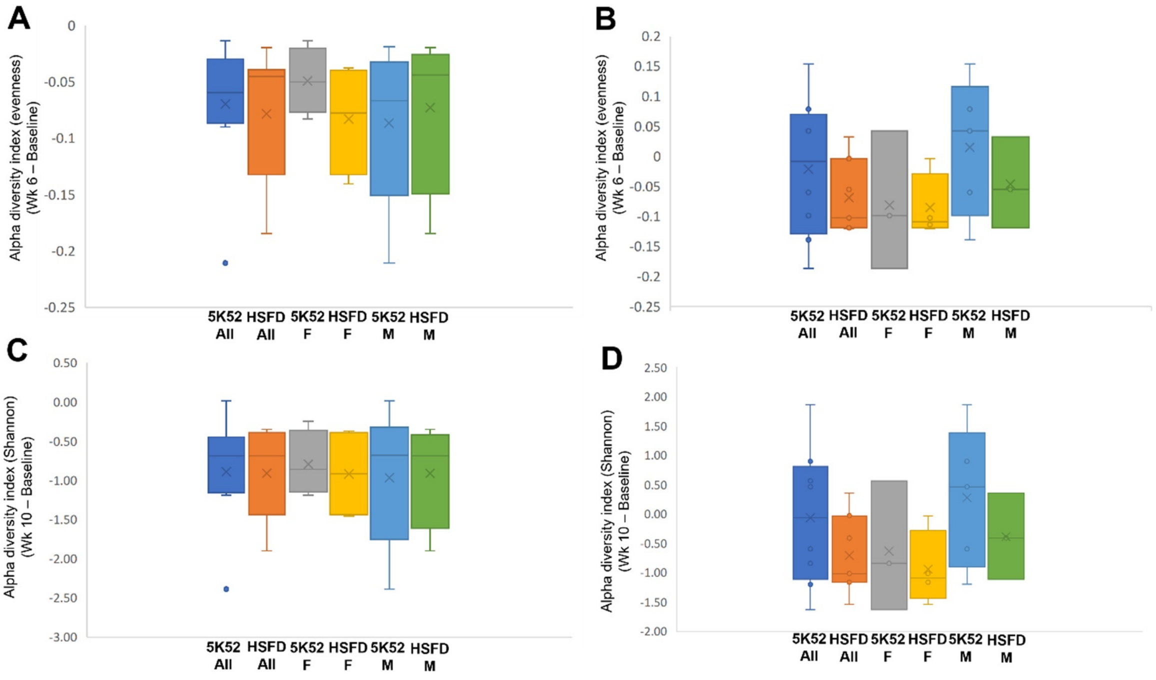

Supplementary Figure S3 Phyla relative abundance in HSFD- and control-fed mice for baseline vs Week 6 (A), and baseline vs Week 10 (B).

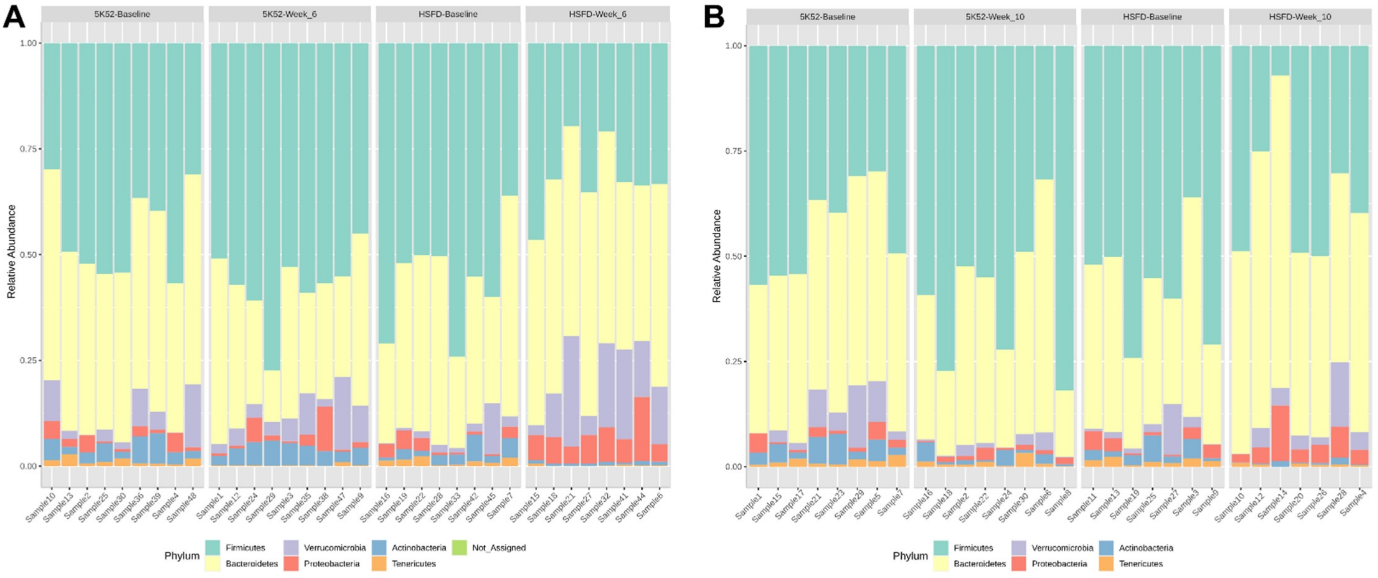

Supplement: Supplementary file 1 [file nutrients-16-01323-s001.zip › Supplementary Materials.pdf]
